# Mothers, Fathers, and Parental Systems: A Conceptual Model of Parental Engagement in Programmes for Child Mental Health—Connect, Attend, Participate, Enact (CAPE)

**DOI:** 10.1007/s10567-016-0219-9

**Published:** 2016-12-02

**Authors:** Patrycja J. Piotrowska, L. A. Tully, R. Lenroot, E. Kimonis, D. Hawes, C. Moul, P. J. Frick, V. Anderson, M. R. Dadds

**Affiliations:** 1 School of Psychology, University of Sydney, Sydney, NSW 2006 Australia; 2 Faculty of Medicine, University of New South Wales, Sydney, NSW 2052 Australia; 3 School of Psychology, University of New South Wales, Sydney, NSW 2052 Australia; 4 Learning Sciences Institute of Australia, Australian Catholic University, Brisbane, QLD 4001 Australia; 5 Department of Psychology, Louisiana State University, 236 Audubon Hall, Baton Rouge, LA 70803 USA; 6 Departments of Psychology and Paediatrics, Murdoch Children’s Research Institute, Royal Children’s Hospital, University of Melbourne, Parkville Campus, Melbourne, VIC 3010 Australia

**Keywords:** Parenting programme, Parental engagement, Co-parenting, Father involvement, Child mental health

## Abstract

Parenting programmes are one of the best researched and most effective interventions for reducing child mental health problems. The success of such programmes, however, is largely dependent on their reach and parental engagement. Rates of parental enrolment and attendance are highly variable, and in many cases very low; this is especially true of father involvement in parenting programmes. This paper proposes a conceptual model of parental engagement in parenting programmes—the CAPE model (Connect, Attend, Participate, Enact) that builds on recent models by elaborating on the interdependent stages of engagement, and its interparental or systemic context. That is, we argue that a comprehensive model of parental engagement will best entail a process from connection to enactment of learned strategies in the child’s environment, and involve consideration of individual parents (both mothers and fathers) as well as the dynamics of the parenting team. The model provides a framework for considering parent engagement as well as associated facilitators and mechanisms of parenting change such as parenting skills, self-efficacy, attributions, and the implementation context. Empirical investigation of the CAPE model could be used to further our understanding of parental engagement, its importance for programme outcomes, and mechanisms of change. This will guide future intervention refinement and developments as well as change in clinical practice.

Pathways to health, well-being and positive social functioning have their roots in childhood. Perhaps the most powerful predictor of these pathways is the quality of early family and parenting environments to which the child is exposed. Relatedly, the best evidence for our ability to positively influence these pathways is associated with programmes that engage and empower parents to create enriching child-rearing environments (for example, Gardner et al. 2006; Sanders et al. 2014). Traditionally, the science of these programmes has largely been concerned with the effects of different parenting strategies on child outcomes, and how best to train and empower parents to enact them. A highly pragmatic but nonetheless important focus has been simply on how to reach parents, have them attend and actively participate in session, and implement these strategies in the child’s environment. Regardless of the quality or content of the programme, its reach into the community is only as good as its ability to engage and mobilise parents; research suggests that a large group of parents experience barriers to accessing such services (Owens et al. 2002).

A focus on parental engagement in treatment programmes has revealed both positive news and difficult challenges. If we take parent training programmes for child behaviour problems as the best researched example (for example, Epstein et al. 2015), it is known that, where available, parent enrolment, attendance, dropout, and implementation rates are highly variable, and in many cases low enough to raise serious questions about the translational reach or community effectiveness of otherwise efficacious evidence-based programmes (Garvey et al. 2006; Heinrichs et al. 2005). That is, while efficacy studies show good outcomes for parents and children, their uptake in population studies is highly variable, in some cases with low rates of attendance (ranging from 37 to 98%), high rates of dropout—over 50% (Friars and Mellor 2007; Kazdin 1996; Wierzbicki and Pekarik 1993), and unclear levels of effective implementation of strategies (Chacko et al. 2016). Recent reviews show that the issue of engagement is particularly critical for fathers (for example, Panter-Brick et al. 2014). That is, compared to mothers, knowledge about their attendance, engagement, and implementation is scarce; where data have been collected, fathers’ engagement is comparatively low. Further, models of engagement that consider parents as a dyad are virtually non-existent.

On a positive note, a recent review of studies testing methods to improve family engagement and retention in child mental health programmes supported the effectiveness of including early engagement discussions, addressing families’ practical and psychological barriers, family systems approaches, enhancing family support and coping, and motivational interviewing within the intervention (Ingoldsby 2010). This review identified some promising approaches to improve engagement (defined as participation and ongoing attendance) and retention (rates of programme completion) but also emphasised that engagement rates are problematically low and deeper understanding of barriers and potential mechanisms is needed.

These recent reviews (Ingoldsby 2010; Panter-Brick et al. 2014) consistently highlight existing challenges in the intervention research with the particular focus on low engagement and retention rates as well as the involvement of parenting systems. The current paper builds upon these reviews by conceptualising engagement as a complex process involving four distinct but interdependent stages, discussing how the engagement model works across family systems and parenting dyads, and offering new insights into engagement conceptualisations and measures. More specifically, this paper critically reviews the literature on parental engagement in evidence-based parenting programmes and explores its conceptualisations and predictors, and the role engagement plays in parenting change and child outcomes. We then propose a conceptual, but pragmatic and testable model of parental engagement in evidence-based parenting programmes—the CAPE model (Connect, Attend, Participate, Enact). To do this, we broadly operationalise parenting programmes as any skills training programme in which parents are empowered to change their parenting in order to produce improved child outcomes. We recognise that the term *parents* is complex and incorporates individual parents, as well as dyadic partnerships and even more complex systems, and thus, we frame our model in terms of individual mothers, fathers, and systemic combinations of these and other caregivers. Finally, while we intend our model to be applicable to any parenting programme as defined above, in order to keep the model grounded and based on the best evidence, we use positive parenting programmes for childhood disruptive behaviour problems as the working exemplar.

The development of the model is presented using the following structure: first, we briefly review evidence that empowering parents to create positive parenting environments is associated with improved child outcomes. Second, we show that the reach of these programmes, defined in terms of recruitment rates is, however, highly variable and that child outcomes are related to parental engagement. Third, we present a model of engagement that describes and organises its components into measureable constructs of Connecting, Attendance, Participation and Enactment by parents at the level of individuals and parental systems. Finally, we discuss how such a model can be used to inform the design, dissemination, and evaluation of future parenting programmes.

## Parenting Programmes and Child and Family Outcomes

A substantial evidence base suggests that parenting programmes based on social learning and cognitive behaviour theories are the most effective interventions to reduce child mental health problems (Eyberg et al. 2008). These programmes, also referred to as ‘parenting interventions’ or ‘parent training’, target parenting skills and parent–child relationships in order to improve child behavioural and emotional outcomes. In these programmes, parents are empowered to increase their focus on positive engagement with the child, reinforce and encourage positive behaviours, and reduce coercive and emotional responses to disruptive, aggressive, and antisocial child behaviour. There are a number of key evidence-based parenting programmes which share a common theoretical basis (i.e. social learning theory) such as the Triple P—Positive Parenting Program (Sanders 1999), the Incredible Years (Webster-Stratton and Years 2011), Parent–Child Interaction Therapy (Eyberg et al. 1995), and Parent Management Training—Oregon Model (Patterson et al. 2002).

Parenting programmes are usually based on a manualised curricula and may involve a range of activities such as discussions, role plays, watching live or video demonstrations of key strategies, and practicing strategies in child interactions within session or during homework tasks (Barlow et al. 2010) in order to learn positive parenting strategies. The standard duration of parenting programmes is typically 8–14 sessions, with weekly sessions lasting 1–2 h, although there are also brief or ‘light touch’ programmes that are less than 8 sessions (Tully and Hunt 2015). Parenting programmes vary widely in their delivery formats and may include face-to-face delivery via individual or group programmes, or self-directed delivery via workbooks or internet based delivery, or a combination of these formats. Parenting programmes also vary in the setting in which they are delivered, including universities, clinics, or community settings such as community centres or schools. Finally, parenting programmes can be delivered as universal or targeted interventions, or as a treatment for children already diagnosed with disruptive behaviour disorders. Some programmes such as Triple P use a public health approach to parenting support which include universal and targeted components to bring about changes in child and parent outcomes at the population level (Sanders et al. 2014).

There is substantial evidence that parenting programmes are effective in the short term in improving parenting skills and a range of childhood outcomes including disruptive behaviour problems—DBPs (Gardner et al. 2006; Gardner et al. 2010; Hanisch et al. 2014; Sanders et al. 2014), autism (Tonge et al. 2014; Whittingham et al. 2009b), and ADHD (Ferrin et al. 2014; Lakes et al. 2011). While there is a lack of research on the longer-term effects of parenting programmes, studies have found that positive outcomes for children are maintained (Sanders et al. 2014), even up to 8–10 years after intervention (Webster-Stratton et al. 2011). Meta-analytic reviews have also found that these programmes can improve a range of psychosocial outcomes for parents such as parental mental health (Barlow et al. 2012; Furlong et al. 2012) and satisfaction with partner relationship (Barlow et al. 2012) that may not be targeted directly within the programme. There is also evidence that parenting programmes are cost-effective and save more money than their delivery costs (Mihalopoulos et al. 2007; Stevens 2014), which provides a worthwhile use of limited health funds.

This line of research and practice considers changes in parenting to be a core mediator in the design of interventions so that strengthening of parenting competencies and improving parent–child interactions can lead to positive child outcomes. Specifically, decreases in dysfunctional parenting (Hanisch et al. 2014), and increases in positive parenting (Gardner et al. 2006), have been found to mediate the effect of intervention on child problem behaviour. Importantly, a recent meta-analysis of psychosocial interventions for child disruptive behaviours found that interventions with a parent component (either on its own or in combination with other components) produced larger improvement in outcomes than child component only or control categories (Epstein et al. 2015). It should be noted, however, that only 45% of studies included in a recent review supported parenting as the primary mechanism explaining improvement in child behavioural outcomes within parenting programmes (Forehand et al. 2014), and the authors called for more research in this area.

Furthermore, some research highlights the importance of not only parenting knowledge and improved skills but also self-efficacy and confidence, and parent–child attributions in improving parenting and child outcomes. Previous literature showed that parenting programmes produce improvements in parental confidence and skill (Gardner et al. 2006) and that parenting knowledge is negatively associated with the levels of dysfunctional parenting (Morawska et al. 2009). Similarly, Dekovic et al. (2010) showed that participation in the Home-Start programme enhanced maternal sense of competence which in turn predicted positive changes in parenting such as decrease in the use of inept discipline and increase in supportive parenting. A number of research reviews showed that group-based parenting programmes are associated with significant improvements in parental confidence (Barlow et al. 2012), and that self-efficacy beliefs relate to parenting practices (Coleman and Karraker 1998) and discipline style (Sanders and Woolley 2005). Specifically, parental self-efficacy and confidence were found to predict changes in parenting so that parents with higher self-efficacy tended to demonstrate more effective and positive parenting (Jones and Prinz 2005; Mouton and Roskam 2014; Spoth et al. 1995). Moreover, some recent research showed that task-specific self-efficacy in responding to disruptive/challenging behaviours and self-efficacy in the parenting role significantly predicted child behaviour (Kirk 2016). Finally, a range of studies reported that parenting programmes can alter parental attributions (for example, Wiggins et al. 2009) but more importantly that parental attributions significantly predict change in dysfunctional parenting, overreactivity in particular (Whittingham et al. 2009a). These factors seem to play important roles in understanding the process of engagement and change in parenting and child outcomes.

Finally, a limited number of studies considered the role of moderators in intervention research in order to identify those who respond differently (i.e. show better or worse outcomes in the events of the same programme being delivered). Moderator research is quite sparse with a limited number of moderators studied and often mixed results. Most available studies focused on sociodemographic variables (child age, gender, socioeconomic status) as potential moderators of intervention outcome. For example, Gardner et al. (2010) reported that boys and younger children, and those with more depressed mothers showed greater improvement in conduct problems post-intervention. The effect of the intervention, however, was not moderated by income, single parenthood, teenage mother, and initial severity of conduct problems. Other studies showed that parenting programme effects vary as a function of the intensity of the intervention, informants (Nowak and Heinrichs 2008), intervention components (Thomas and Zimmer-Gembeck 2007), or initial problem scores (de Graaf et al. 2008). Moderation analyses in the literature, however, showed mixed results. For example, McGilloway et al. (2012) showed that the intervention effects on the primary child outcomes were not moderated by child or family demographic characteristics or risk factors such as age, gender, being at risk of poverty, socioeconomic disadvantage, and risk factors for behavioural problems.

More importantly, there is paucity of theoretical models of moderators in parenting interventions (Gardner et al. 2010), and potential moderators affecting the implementation of skills and strategies learned in a programme have been omitted in the literature. One study showed that child’s gender, family income, family type, pre-intervention parental stress did not moderate parents’ capacity to change their dysfunctional parenting (i.e. sociodemographic and family variables do not seem to compromise parents’ ability to change their practices) (McTaggart and Sanders 2007). Once again, the focus has remained on sociodemographic variables.

We propose there might be other factors moderating the change in parenting and implementation of positive parenting strategies that have not been explicitly studied so far. For example, family chaos conceptualised as a measure of home confusion and disorganisation has been shown to relate to less effective parental discipline and elevated behaviour problems (Dumas et al. 2005) which suggests that family chaos could interfere with the process of parenting change. Similarly, previous research showed that the effects of parenting interventions are compromised when parents cannot work as a team to implement the programme (Dadds et al. 1987), highlighting the importance of co-parenting for treatment success. It is important to consider family context variables that can interact with the implementation of learned strategies and thus affect child outcomes.

Research discussed in this section has been primarily or exclusively conducted with mothers, and relatively little is known about parenting programmes’ reach with regard to fathers. The next section offers an overview of research on father involvement in parenting programmes and highlights substantial gaps in the current literature.

## Fathers in Parenting Programmes

The importance of including fathers in parenting programmes has been continuously highlighted in the last decade (Lundahl et al. 2008; Panter-Brick et al. 2014; Pfitzner et al. 2015). The substantial evidence base supports the important role of fathers for children’s development, and yet, the research on father engagement in parenting training is limited and findings often inconclusive. Fathers remain underrepresented across parent intervention studies, and when included, researchers rarely assess the independent effect of father involvement and the fact that they may play different and/or complementary roles to mothers (Tiano & McNeil, 2008). A systematic review on father involvement in behavioural parent training for ADHD showed that the majority of studies include mothers only as both participants and raters of children’s outcomes, and none of 32 included studies addressed the independent effect of father involvement (Fabiano 2007). More recently, Panter-Brick et al. (2014) conducted a large-scale systematic review of 199 publications investigating father inclusion in a range of parent interventions, as well as preventive programmes related to prenatal health, alcohol abuse, and maltreatment. Their review confirmed that only few intervention evaluations reported data on fathers or couple effects with most studies focussing on mothers and as a consequence, rates of father involvement are difficult to estimate. Where reported, these rates tend to be low, ranging from 13 to 21% (Fabiano 2007; Scourfield et al. 2014).

The lack of research or limited inclusion of fathers is even more striking in the light of a meta-analysis which showed that studies that had included fathers (in contrast to those that had not) reported significantly more positive changes in children’s behaviours (*d* = 0.48 vs. *d* = 0.20) as well as better parenting practices (*d* = 0.54 vs. *d* = 0.06) immediately after the training (Lundahl et al. 2008); these significant differences were not maintained at follow-up. Other researchers, however, indicated that fathers may play an important role in maintaining the intervention effects over time. For example, Bagner (2013) argued that father involvement may lead to increased parental consistency at home, which then leads to maintaining the intervention effects. Similarly, Panter-Brick et al. (2014) suggested that behavioural change is unlikely to be sustained when only one parent is targeted in the intervention, highlighting the need of a parenting team engaged in a programme.

Despite the general consensus in the literature on the importance of father involvement in parent training, not much is known about the most effective ways to achieve it. A range of reviews have highlighted potential barriers to father involvement. These may include lack of awareness, perceiving disruptive behaviours as less problematic, time (i.e. work commitments), female-oriented services, low organisational support (e.g. lack of father-focused policies, incompatible working hours), or lack of information about the content of such programmes (Bayley et al. 2009).

Father engagement is a complex and multidimensional construct, and to this date, there is very little research indicating which factors are most important in getting fathers engaged (Pfitzner et al. 2015). Much research will need to be done to address this gap in the literature. Specifically, Tiano and McNeil (2008) emphasised the need for multimethod assessments, including fathers in the assessment process, and developing father-focussed measures acknowledging that fathers interact with children differently and may play different roles. For example, when both parents are included in a programme, different aspects of parenting may change for mothers vs fathers as a result of their participation, and some research suggests that mothers’ benefits are greater than fathers (for example, Fletcher et al. 2011). It is also possible that mothers play an important role in father involvement and act as gatekeepers. Previous research showed that maternal characteristics and beliefs about the role of the father predicted father involvement in child rearing (for example, McBride et al. 2005); maternal regulation of father involvement in parenting programmes has not been explored yet and these issues are further discussed when considering the systemic context of the parental engagement model (CAPE).

We recognise that parenting teams may include many different caregivers; the current model, however, focuses on mother–father parenting teams and their engagement in parenting programmes. Parental engagement and the CAPE model articulating a set of factors hypothesised to play important roles in parental engagement and programme effectiveness, and highlighting the importance of a parenting team is now discussed.

## Parental Engagement

The remarkable efficacy of parenting programmes is tempered by their limited reach, often operationalised as the percentage of study participants recruited from the target population of eligible participants (i.e. recruitment or enrolment rates), and lack of sustained attendance and active participation. The limited reach and impact of such programmes means that help and support is not provided and public health resources are not efficiently used.

Engagement in parenting programmes has been continuously identified as a crucial step to intervention success and the quality of mental health treatment (Haine-Schlagel and Walsh 2015). However, there is currently no agreement as to the definition of engagement and lack of uniformity in reporting engagement rates, with previous research highlighting the need for more systematic approach to such conceptualisations (Chacko et al. 2016; Staudt 2007). A range of definitions have been offered identifying levels of engagement such as from the initial reach of a programme to subsequent completion (Morawska and Sanders 2006); recruitment and retention (Axford et al. 2012); attendance, adherence, and cognitive preparation (Becker et al. 2015); all stages from help-seeking, attendance to following through with home action plans (Haine-Schlagel and Walsh 2015); enrolment, attrition, attendance, within-session engagement, and homework completion (Chacko et al. 2016); or intent to enrol, enrolment, and retention (McCurdy and Daro 2001).

These reviews largely support the conceptualisation of engagement as a multidimensional construct identifying multiple stages, levels, and domains (Becker et al. 2015). Most commonly identified stages include recruitment/enrolment, attendance, and retention of parents. However, what often remains overlooked in the existing literature is the process of engagement, relationships between different stages, how engagement relates to parent and child outcomes, and the role of the dyadic context in parental engagement. Moreover, data on within-session engagement and homework completion are rarely reported (Chacko et al. 2016), and corresponding active participation is often omitted from definitions of engagement. The CAPE model described here addresses these limitations by presenting a comprehensive process model of parental engagement including four main stages: Connect, Attend, Participate, and Enact. It builds on the previous literature by identifying enrolment and attendance stages (Connect and Attend), but also conceptualises active participation and enactment of learnt strategies as two additional dimensions relating to parental engagement. Moreover, the model presents engagement as a process, discusses its effects on parent and child outcomes, and potential mechanisms, and considers parental engagement in the context of complex family systems.

## A Model of Parental Engagement

**Fig. 1:**
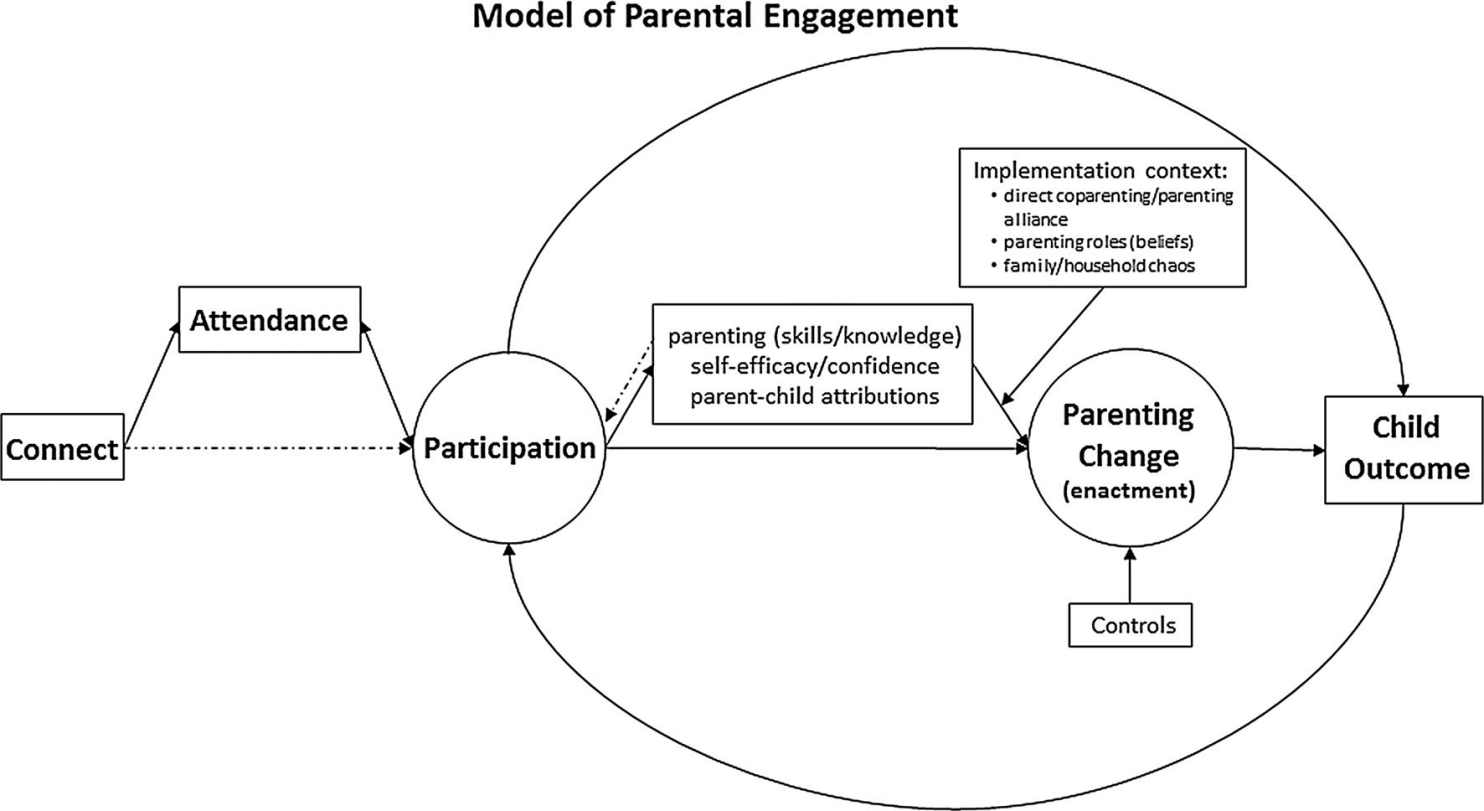
Model of parental engagement

It is increasingly highlighted that significant positive outcomes cannot be achieved without active and meaningful participation by parents, which is often operationalised as paying attention, being receptive and open to new ways of interacting with children, actively contributing to discussions and tasks, completing homework tasks or asking questions. Research has showed that active participation is associated with improved parenting, including increased supportive/positive parenting and reduced inconsistent and negative parenting (Baydar et al. 2003). Previous reviews defined engagement as recruitment—attracting parents, retention—sustained attendance, and involvement—extent to which parents participate, or put simply participation and ongoing attendance (Ingoldsby 2010). Building on these definitions, the CAPE model of parental engagement (Fig. 1) consists of recruitment/enrolment (Connect), retention (Attend), involvement (Participate) but also includes implementation of newly learned strategies and techniques (Enact). Each of these stages and their relationships are now discussed.

The first stage of the model—Connect, refers to the reach of the programme and connecting with parents and their decision to enrol. Attendance refers to continuous presence at sessions or logging in (in case of online programmes)—i.e. perseverance. Programme Participation, however, remains elusive of definition, and its operationalisations vary greatly across the studies. In this paper, we refer to Participation as a set of actions that go beyond Attendance at the session, such as home practice completion or active group discussion which are believed to enhance the proximal intervention outcome, which is parenting change. Despite the fact that these two constructs remain strongly related, their relationships with a range of predictors and outcomes may differ.

Specifically, the first three model stages (Connect, Attend, and Participate) are known to be predicted by a set of factors including family characteristics (e.g. parent’s age, socioeconomic status, economic stress, family structure), child characteristics (e.g. age and gender, difficulties profile), family processes (e.g. parental mental health, interparental conflict and relationship quality, family/household chaos, and the current level of parenting skills), contextual factors (e.g. beliefs about parenting roles, cultural factors, parental personality, and help-seeking beliefs), and organisational factors (e.g. therapist factors, programme help interface, access and availability factors). These factors represent a range of perspectives and contexts often included in ‘barriers to treatment’ models (Nock and Ferriter 2005) that may influence parents’ will and ability to engage in a parenting programme and have been extensively studied (Kimonis et al. 2014; Snell-Johns et al. 2004).

To the best of our knowledge, only one study has explicitly investigated the potentially differential effect of family and child factors on Attendance and Participation. Nix et al. (2009) in their study with parents of children with severe conduct problems showed that a range of variables such as lower education, stressful circumstances, and severity of child’s behaviour problems predicted the quality of parent participation, but not their attendance. Other research suggests that family-related, pragmatic, organisational, and scheduling factors (i.e. organisational, scheduling) play a greater role in the initial stages of parental involvement, whereas the strength of neighbourhood networks is important at the later stages—i.e. participation and completion (Eisner and Meidert 2011). This line of research highlights the importance of differentiating when parents Attend from when they actively Participate and commit themselves to a parenting programme.

Considering these constructs separately is also important in the light of potentially varying outcomes or their contribution to positive changes in parenting and child outcomes. The CAPE model proposes that despite the immense importance of Connecting with parents and encouraging their Attendance, it is active Participation that has the greatest impact on parenting. To the best of our knowledge, no research to date has explicitly tested whether Attendance and Participation may independently lead to similar parenting outcomes. Some previous research, however, indicates that it is the programme Participation that has substantial effect on outcomes in the context of parenting programmes. For example, Baydar et al. (2003) showed that greater parent programme engagement, operationalised as higher rates of attendance, homework completion, and involvement in group discussion, was associated with better parenting outcomes. They showed that a higher level of engagement significantly reduced harsh/negative and inconsistent parenting, and increased positive/supportive parenting.

We propose that active Participation has a major effect on implementation of positive parenting strategies (Enactment). We also emphasise the correlational relationship between Attendance and Participation so that changes in Attendance are likely to be associated with changes in active Participation.

Finally, we argue that two potential types of Participation should be recognised, namely *direct* and *indirect* Participation. Direct Participation refers to active commitment, physical presence, and involvement with the programme materials, whereas the latter refers to acquiring information from other sources especially in the context of a parenting team, i.e. one parent may register and directly engage with a programme (in person or online) and later teach and train their partner in the use of relevant strategies. This idea is represented in the CAPE model (Fig. 1) by a dashed line from Connect to Participation which suggests that Connection may influence (indirect) Participation directly without Attendance. To our knowledge, no available research has considered information sharing at the family level as an important factor in parenting programmes. We believe these two types should be reflected in the engagement measurement models in future studies in order to further our understanding of the importance of active Participation as well as co-parenting in the context of parenting programmes.

The major focus so far has been on active Participation which is thought to mediate the effect of the programme on the Enactment of positive parenting strategies included in the process model of engagement. The question, however, remains how active Participation produces these positive changes in parenting and what factors can affect this process. Specifically, parent training is largely implemented as an intervention to improve child behavioural outcomes, and the process conditioning positive changes needs to be further explored.

## A Process Model of Engagement and Parenting Change

This section takes a closer look at the CAPE model and outlines a process model of the relationship between parental active involvement and change in parenting, i.e. Enactment of newly learned parenting strategies and techniques, especially in response to child’s behaviour in a home/family context (Fig. 1). We argue that parent Participation is the key mechanism to positive change in parenting and as such, it remains the primary focus of this model. Similarly, the sustained and competent Enactment of the taught parenting principles is the critical causal mechanism in the programme theory of parent training (Eisner and Meidert 2011) and is discussed here accordingly.

As outlined previously, parent Participation is a latent factor measured by a set of indicators such as involvement in discussions or homework completion, which importance for therapy outcome has been highlighted (Kazantzis et al. 2000). For the simplicity of the model presentation, we do not include example covariates in the figure; these are discussed in the text instead. As previously mentioned, little is known about the mechanisms of change from programme Participation to Enactment, and the following model presents some hypothetical mechanisms explaining parenting changes and outlines what other factors may play a role in this process considering the role of a parenting team.

Figure 1 shows the elaborated model specifying the putative mechanisms influencing relationships between Participation and Enactment of positive parenting strategies. Firstly, three potential mediators of the relationship between Participation and parenting change (Enactment) were introduced, namely parenting skills/knowledge, self-efficacy and confidence, and parent–child attributions. *Parenting skills* and *knowledge* refer to acquired information about parenting, awareness of a range of parenting strategies, and ability to implement such strategies. *Self*-*efficacy* and *confidence* focus on parental sense of confidence and belief in one’s own ability to enact various parenting strategies. Finally, *parent*–*child attributions* refer to parents’ thoughts/beliefs about child mental health problems and associated behaviours. Previous research showed that parenting programme can produce improvement in these three mediators (e.g. Gardner et al. 2006), which are in turn associated with changes in parenting (e.g. Sanders and Woolley 2005; Whittingham et al. 2009a).

These factors may therefore be the key mechanisms by which parenting programmes elicit Enactment of parenting strategies. We hypothesise that parenting programmes positively affect these factors which in turn, predict the change in positive parenting, and contribute to successful Enactment of newly learned strategies. It is also possible that the relationship between Participation and these mediators may be reciprocal (as indicated by a dashed line in Fig. 1), so that participation-induced change in skills, confidence levels, or in parent–child attributions may lead to changes in future Participation. Specifically, increase in skills or perceived self-efficacy can either positively affect further participation so that parents see initial changes and choose to continue to maximise potential benefits, or can have a negative effect where parents lower their levels of participation as they feel that all the goals have already been achieved; these hypotheses are in need of further investigation.

Furthermore, it has been previously argued that effective interventions produce a sequence of change in families, social interactional patterns in particular (Patterson et al. 2010). This highlights the need to consider process models and intervention mechanisms in the context of family systems. Consequently, the model was extended to include the *implementation context* which is thought to moderate the pathway from parenting skills, confidence, and attributions to Enactment. The implementation context includes three factors: *parenting alliance* (i.e. direct co-parenting), which refers to an existence of a parenting team where both caregivers are involved and consistent in enacting relevant strategies; *beliefs about parenting roles*, that is beliefs about the importance of mother and father involvement in parenting; and *family/household chaos* (i.e. noise, lack of routine and order). As previously discussed, there is paucity of research considering potential moderators of parenting programmes. Where available, studies focus on demographic factors and moderators of the relationship between participation and child outcome, rather than the implementation of newly learned strategies (Enactment). We propose that the level of these potential moderators can significantly affect the mechanism of change; that is, even when the positive change in mediators is achieved as a result of parent Participation, it may not have a desirable effect on parenting (and the distal outcome—i.e. child behaviour) due to lack of parenting alliance, high levels of chaos, or undermining parenting roles.

As noted, more often than not parenting programmes are implemented to address child behavioural problems. It is therefore important to consider in the current model the distal outcome—positive changes in child behaviour. The CAPE model can be further extended to include child outcomes as presented in Fig. 1. Following previous research, we argue that positive changes in parenting (Enactment) will likely lead to improvements in child behavioural outcomes. More importantly, however, there may exist a feedback loop between parent Participation and child outcomes. Specifically, parent Participation in a programme may maintain some direct effects on child outcomes, and positive improvements may in turn encourage further parent Participation. For example, when parents recognise the change in their own parenting and observe positive changes in their child’s behaviour, the level of their overall engagement is likely to change due to perceived effectiveness of a programme. Or alternatively, parents may decide to dropout of the programme or minimise their Participation due to the perception that no changes have been made, or alternatively that all the positive outcomes have already been achieved. In summary, we believe this feedback loop may have positive or negative effect on child outcomes and parent Participation but no research to date has explored these associations and future studies will need to explore the interdependence of parent Participation and child outcomes, as well as the importance of family systems in these processes.

### Systemic Context

Contemporary models of developmental psychopathology emphasise a focus on dynamic family systems as well as individuals. This systemic focus may be particularly important for models of parental engagement for four reasons. First, there is a wealth of evidence to show that the development, maintenance, and treatment of children’s behavioural problems are often associated with broader family problems such as marital or interparental discord (Gable et al. 1992; Mathijssen et al. 1998). Importantly, the efficacy of parenting interventions and thus the positive outcomes for children are compromised when parents cannot work as a team to implement the programme. Interparental conflict seems to be linked with poorer parental engagement, higher dropout, and poorer implementation of parenting techniques (for example, Prinz and Miller 1994).

Second, perceptions of the need to seek help for child DBPs, awareness of available parent training programmes, and the decision to approach and engage with a parenting programme will vary between parents. It is possible for the engagement process to be initiated by one parent and then communicated to the other, who then may choose to follow-on and participate. This process is still likely managed by mothers as primary caregivers with fathers playing secondary roles. The reasons for this may be driven by family roles that are reflective of more broadly engrained cultural values (Humenick and Bugen 1987) through to simple practical issues like parental availability during service opening hours, the need for child care and so on.

Third, engagement by individual parents may not be fully captured by simplistic indices like attendance. For example, one parent may attend the programme but convey the information and skills learned to the partner at other times. Thus, a comprehensive model of parental engagement will need to conceptualise and measure how information and skill development is delivered to and moves through the parental system which is likely to be affected by the quality of the relationship and individual factors.

Consequently, we propose that consideration of interparental dynamics is central to a model of parental engagement. Interparental or systemic aspects of parental engagement have, however, rarely featured in the engagement literature despite substantial methodological progress and state-of-the-art techniques that help to answer questions about family processes and interactions (Cook 1994). In this section, we discuss all four aspects of the parental engagement model (Connect, Attend, Participate, Enact) in the systemic context.

At the first stage of the model—Connect, parents learn about a programme either as a result of promotional efforts or it may be recommended to them by a friend or a professional. Parental access/recruitment and enrolment, however, may be affected by interparental processes. These may include practical issues such as scheduling and child care if both parents are attending the programme, but also issues such as gatekeeping. Gatekeeping refers to regulating one parent’s involvement by the other parent and has not been explicitly studied in relation to parenting programmes. Previous research, however, showed that mothers in particular and their beliefs/perceptions about the importance of father involvement in children’s lives as well as satisfaction with father involvement are associated with the frequency of father involvement in children’s lives (De Luccie 1995; McBride et al. 2005). It is unclear, however, whether mothers regulate fathers’ enrolment in parenting programmes. Some promotional materials attempt to indirectly access fathers through mothers, and practitioners and researchers report that mothers may encourage fathers to sign up and complete a parenting programme.

Previous research showed that maternal beliefs/perceptions about the importance of, and satisfaction with, father involvement are associated with the frequency of actual father involvement in children’s lives (De Luccie 1995; McBride et al. 2005). This highlights the importance of systems and ecological approaches where interactions between father and a child might be affected by other dyadic events and processes in the family system—for example, mothers’ expectations, attitudes, and/or behaviours. In the light of regulatory roles that mothers seem to play, it is important to consider whether they also play a role in influencing father engagement in parenting programmes. To our knowledge, maternal regulation of father involvement in parenting programmes has not been explored yet, however, previous research suggest that the attitudes of mothers may impact on fathers’ participation in parenting programmes (Glynn and Dale 2015). This is particularly important in the light of evidence that father participation in parenting programmes leads to greater reductions in child externalising behaviours and greater improvements in parenting (Lundahl et al. 2008). Much less is known about paternal gatekeeping and the role fathers play in accessing parenting programmes; future research will need to address this gap.

Similarly, parental gatekeeping and scheduling may play a role at the second stage of the model—Attend. An extensive research base suggests that practical difficulties such as lack of transport and/or child care, inconvenient times, and work commitments are the common barriers to parents’ attendance (Snell-Johns et al. 2004; Spoth and Redmond 2000), fathers in particular (Bayley et al. 2009; Snell-Johns et al. 2004; Spoth and Redmond 2000). Despite the efforts to reduce these barriers at the service level (for example, Dumka et al. 1997), mutual support and marital consensus seem crucial to programme completion for two parent families. What is more, it is likely that marital discord/interparental conflict can affect parental help-seeking attitudes and consequently, reduce parental attendance and contribute to dropout.

Following the literature already reviewed, the CAPE model highlights the importance of active Participation and its association with better outcomes. Once again, it is not only about recruiting parents and encouraging them to attend and persevere but also about encouraging active participation in the programme. Researchers often operationalise active participation (or engagement) as involvement in discussions and homework completion, but do not consider the contribution of the family systemic context. The CAPE model differentiates between direct and indirect Participation by allowing one parent to attend the sessions but both parents to actively participate. In other words, the parent who attends the programme may discuss the content of the programme with their partner, they may teach their partner new strategies, and they may both complete homework. This highlights the role of a ‘parenting team’ where both parents are involved, remain consistent in their parenting, and aim to achieve the same goals.

Importantly, however, such indirect Participation may have negative consequences when transferred information is inaccurate or purposely modified by the attending parent which may affect the parenting balance. No research to date has examined the effect of both parents’ active participation on child outcomes but indirect evidence comes from a review on father involvement in parent training, which showed that studies that had included fathers reported significantly more positive changes in children’s behaviours and better parenting practices than those that did not (in the short-term), supporting the importance of a ‘parenting team’ (Lundahl et al. 2008); this meta-analysis, however, did not examine active participation. Finally, one study exploring father perceptions and barriers to men’s engagement with parenting support programmes found that their engagement with support services was largely affected by the lack of recognition and the presumption of professional staff that they were ‘secondary’ or part-time caregivers (Cosson and Graham 2012). This represents an indirect maternal gatekeeping through cultural emphasis on mothers as primary carers and disabling fathers from playing primary or at least equal roles in parenting.

Lastly, marital consensus and being a part of a ‘parenting team’ is crucial in the final stage of the mode—Enact. The key to successful intervention is in applying newly learned strategies consistently across time and situation but also between parents. Previous exploratory research showed that the implementation of child management strategies aiming to reduce child behavioural problems can be improved by decreased parent-to-parent aversive behaviour and marital discord as a result of additional partner support training (Dadds et al. 1987a). Similarly, Dadds et al. (1987b) found that partner support training produced significant changes in child behaviour at 6-month follow-up among families experiencing marital discord, showing that inclusion of brief marital intervention may help overcome the relapse/treatment failure for maritally discordant families. These studies support the interdependence of marital relationship and children’s disruptive behaviours. Finally, some previous research suggests that father involvement is particularly important in the maintenance of parenting programme effects (for example, Bagner and Eyberg 2003; Webster-Stratton 1985). Researchers have suggested that these findings point towards parenting consistency at home and parents acting as a team. However, one study found that mothers participating in a parenting programme experienced difficulties when implementing the new techniques which included gaining the support of their partner, changing established patterns of parenting, and finding the time to parent together; and discrepancies in parenting techniques seemed to lead to parental conflict (Mockford and Barlow 2004). This line of research emphasises the importance of the family systemic context which is yet to be thoroughly studied in relation to parenting programmes.

This brief overview of literature on the interdependence of parental relationships and children’s behavioural problems and the importance of marital relationship in the context of parenting programmes emphasises that parental engagement needs to be considered in the interparental context. Each aspect of the model, from Connecting to Enactment, does not happen in isolation, and the role of both parents, where applicable, and their interactions need to be included in all engagement models. This model should further guide developments in the area with the particular focus on the role of family systems. It is expected that the conceptual model will be adapted to a measurement model and tested in a range of settings (university, clinics, and community) with diverse populations and across a range of delivery modalities included individual, group programmes, and online programmes. The proposed measurement plan for the CAPE model is discussed in the next section.

### Measurement Model Testing

The veracity and applicability of conceptual models such as the CAPE model need to be tested in the relevant social context (Dekovic et al. 2012), and such tests are likely to further inform the development of these models. This section discusses how each part of the CAPE model can be adapted to a measurement model, how relevant stages of the model can be measured, and what issues need to be considered when testing it in a range of settings.

It is important to consider how to operationalise and measure Connecting, Attendance, Participation, and Enactment of positive parenting strategies. The Connection stage relates to the reach of a programme and recruitment/enrolment rates. These can be calculated at the population- or sample-level, and illustrate the proportion of participants who took part in the study out of all possible participants (e.g. parents of children with DBP aged 0–18) at the population level, or out of all contacted/approached people at the sample level. These rates play an important role in evaluating research quality, effectiveness of recruitment strategies, and the reach of a programme; yet, they remain often omitted in research reports.

Furthermore, we believe it is crucial to differentiate between Attendance and active Participation in a programme. This will allow us to study individual contributions of Attendance and Participation to proximal and distal outcomes. As previously mentioned, attendance may be measured by presence at an assessment session (if available), proportion of sessions attended, or online modules unlocked (in case of an online programme). These indicators and measures are dictated by programme specificity and data availability. On the other hand, records of homework completion and involvement in discussions, and group facilitator ratings of engagement can be used as indicators of parent Participation. To the best of our knowledge, no psychometrically valid and reliable scales of parental engagement have been developed. However, previously mentioned indicators have been used to form ad hoc scales of active Participation (for example, Baydar et al. 2003). Once again, the number and type of participation indicators are largely programme specific. For example, a component of PCIT is home practice of skills by parent in 5–10 min play scenarios, which are recorded on homework sheets.

Importantly, measures of indirect Participation (i.e. information sharing) are also encouraged. This concept outlined in the CAPE model relates to the family system context and how both parents can actively participate and benefit from a programme without attending the sessions. Ad hoc measures of partner information sharing could involve questions regarding parental communication and discussion over the programme content or reviewing programme materials provided by a partner. Similarly, our model emphasises the potential role of gatekeeping at various stages of parental engagement and future studies should aim to include relevant measures. One example would be the Role of the Father Questionnaire (ROFQ; Palkovitz 1984) which measures the extent that a parent believes the father’s role is important to child development. Even though this measure is not specific to parenting programmes, we believe it could act as a proxy measure for gatekeeping attitudes (and behaviours).

The model also calls for measures of the family systemic context at the attitude- as well as behaviour-level. Such measures should focus on marital consensus, satisfaction, and cohesion, for example, the Dyadic Adjustment Scale (DAS; Spanier 1976) or coding observations of maternal and paternal behaviours using the System for Coding Interactions in Dyads (SCID; Malik and Lindahl 2004). The proposed move towards systemic relations and the importance of family systems in parenting programmes also emphasises the role of father engagement in parenting programmes.

Finally, child and parenting outcomes remain the focus of intervention programmes and their relative change over time is often used as an indicator of programme effectiveness. Consequently, reliability of such measures as well as their sensitiveness to change is of crucial importance. A range of child behaviour measures has been used in parenting programmes research from clinical interviews to psychopathology screening measures. The most commonly used measures include the Eyberg Child Behavior Inventory (ECBI; Eyberg and Pincus 1999), the Strengths and Difficulties Questionnaire (SDQ; Goodman 1997), the Child Behaviour Checklist (CBCL; Achenbach and Rescorla 2001), or the Diagnostic Interview Schedule for Children (DISC; Shaffer et al. 2000).

On the other hand, measures of parenting are less consistent and often criticised for their low psychometric standards. A recent review of the psychometrics of parenting measures showed that the majority of parenting assessment measures do not provide high-quality psychometric data and commonly lack scoring procedures and norms (Duppong Hurley et al. 2014). Nonetheless, commonly used questionnaire measures of positive involvement with children, supervision, discipline strategies, and consistency include the Alabama Parenting Questionnaire (APQ, Frick 1991), Coping with Children’s Negative Emotions Scale (CCNES; Fabes et al. 1990), or the Parenting Scale (Arnold et al. 1993). More importantly, however, there is need for reliable measures of implementation of positive parenting strategies, i.e. whether and how effectively parents enact newly learned strategies (Enactment). These would be largely based on self-report parent measures of parents’ use of parenting strategies, and home/clinic observations of specific parent–child interactions. The Enactment measures need to focus on parents’ ability to respond to child’s positive and challenging behaviours which goes beyond practicing new strategies. This also emphasises the importance of multi-informant approach and use of observational measures which are discussed below.

The choice of informants in mental health research is a subject of a long-lasting debate with cross-informant correlations of .30 being common between separate raters of children’s behaviour problems (Achenbach et al. 1987; De Los Reyes et al. 2015). For example, Rubio-Stipec et al. (2003) have demonstrated discrepancies between parent and child reports of depressive and disruptive symptoms. Similarly, discrepancies have been found between parent and teacher reports, with a suggestion that they may be a function of socioeconomic and demographic factors such as income or maternal age (Lederberg Stone et al. 2013). Importantly, previous research also suggests that discrepancies exist between mothers and fathers as informants (Dave et al. 2008; Langberg et al. 2010), and such disagreements may be a function of parent–child relationship and parental psychological symptoms (Treutler and Epkins 2003). This highlights the importance of including not only multiple informants in research designs but also observational and objective measures that can be later used to validate available self-report data. Also, such models should be tested separately for mothers and fathers attending parenting programmes.

Finally, the CAPE model touches upon mechanisms underlying intervention effects. Understanding mechanisms cannot be a matter of one study but rather a programme of research which would allow evidence accumulation from different types of studies and from across different disciplines (Kazdin 2007). This also calls for the use of the start-of-the-art methodologies and analytical tools. However, in the first place, data on a range of mediators and moderators must be collected and available to allow the analysis of process models and the change they produce as outlined in this paper.

## Discussion

There is increasing evidence showing the efficacy and effectiveness of parenting programmes and interventions for a range of child mental health problems, and behavioural difficulties in particular. Positive changes in parenting remain the most likely mechanism producing improvement in a range of child outcomes. In order to achieve this change, parents need to Connect, Attend, and Participate in a programme, and Enact the strategies taught. These four interdependent stages are conceptualised as parental engagement. The CAPE model provides a conceptual framework for considering engagement beyond simple parental attendance, to an expanded model of engagement that considers an ongoing process of connection and enactment that occurs across family systems and parenting dyads. It offers a broad framework that can shape our understanding of parental engagement and its importance in the process of change that can inform research designs of future studies.

Consideration of the multiple factors outlined in the CAPE model may help ensure that strategies aimed at improving parental engagement and parenting change take into account primary mechanisms and potential moderators such as parenting alliance or perceptions of the parenting roles. The process model suggests three potential intermediate variables including parenting skills/knowledge, self-efficacy, and confidence, and parent–child attributions. All these variables have been previously linked to changes in parenting within parenting programmes. However, they remain to be formally tested as mechanisms of Enactment. This also highlights the need for well-designed, methodologically advanced studies, and randomised control trials. Specifically, such studies will help to assess the roles of Attendance and Participation, relative contribution of family, child and organisational factors, the effects of engagement on parenting change (Enactment), and the effects of parenting on child outcomes. This will then facilitate research into mechanisms of therapy and guide future efforts to optimise therapeutic change.

As previously argued, the focus should remain on family systems and dyadic engagement of both parents (where available) which will further enable the study of parallel mechanisms for mothers and fathers as well as family dynamics. Importantly, however, much effort will need to be put into normalising father involvement in parenting programmes and appreciating the diversity of mother and father roles. Previous research suggests that fathers may benefit less than mothers (Fletcher et al. 2011) and more research is needed to investigate why this is the case. This may be a consequence of basing these programmes on mothers’ needs and validating them with female samples. It is possible that mother and father needs and preferences towards parenting programmes differ, but it is also possible that mechanisms proposed in our models differ between mothers and fathers. Acknowledging the differences between parent informants and collecting assessment data from both mothers and fathers seem crucial steps to further developing this research area as well as increasing father engagement (Pfitzner et al. 2015). Importance and involvement of fathers in parenting continuously increases as many societies move towards more co-parenting styles, and parenting programmes need to focus on family systems. It is, however, important to acknowledge that parenting teams are not limited to mothers and fathers and can include many different caregivers such as grandparents or extended family members. The current model focuses on parental engagement in the context of mother–father co-parenting, and its applicability and relevance to extended and/or intergenerational families, same-sex couples as well as parenting programmes that are designed to address physical difficulties rather than mental health problems will need to be further explored.

Finally, the CAPE model and its grounding in available research evidence encourage its implementation into clinical practice. The particular focus should be on the role of parental engagement and associated barriers and challenges. Previous models and research suggests a range of strategies to reach parents and mitigate attrition risk factors which need to be considered at both individual and organisation levels (Watt and Dadds 2007). Our review suggests engagement strategies should place increasing focus on engaging fathers and parents within the context of the parental system, at the level of individual therapist behaviour, programme delivery, and the policy level. Thus, improvements in engagement will require targeting the clinical–interpersonal skills of practitioners, programme and needs level organisation of clinics and service agencies, and policy-level change to reflect the prioritisation of engagement strategies for both mothers and fathers conceptualised within flexible parental systems. However, no data are available at this stage to assess whether these strategies are effective and assessment of previously discussed strategies should be a research priority. At the level of programme and policy change, even simple changes like extending opening hours of clinics, increasing the size of consultations rooms to work with couples, improving child care arrangements so both parents can attend, and making clinics appealing or at least not alienating may make worthwhile changes to engagement rates and thus child outcomes.

The CAPE model also highlights the importance of acknowledging the role of parental confidence and skill, their child attributions as well as family environment and parenting alliance in treatment success. These factors should be incorporated in treatment strategies to support the maintenance of intervention effects. For example, clinicians and practitioners should address parental conflict and lack of consistent use of parenting strategies. We propose that the current model can be applied to a range of therapeutic efforts and intervention types including individual and group programmes as well as clinic-based and community interventions. The conceptual model of different stages of engagement and processes involved remains largely the same across these varied delivery formats with some changes required to the measurement model such as indicators of active participation (e.g. group discussion).

The presented model offers a general conceptual framework for the study of parental engagement and change in parenting, and remains to be studied empirically. This paper aimed to shape our thinking about the wide context of parental engagement and how it should be approached at both research and clinical levels. The evidence in this area is sometimes limited, and the proposed framework serves to guide researchers towards further exploration of mechanisms underlying parental engagement as well as factors that can affect these processes. It is hoped that investigation of proposed models in a range of settings can further our understanding of parental engagement, its importance for programme outcomes, and mechanisms of change, which will in turn guide future intervention developments as well as change in clinical practice.
